# Genomic analyses reveal evolutionary and geologic context for the plateau fungus *Ophiocordyceps sinensis*

**DOI:** 10.1186/s13020-020-00365-3

**Published:** 2020-10-06

**Authors:** Jie Liu, Linong Guo, Zongwei Li, Zhe Zhou, Zhen Li, Qian Li, Xiaochen Bo, Shengqi Wang, Junli Wang, Shuangcheng Ma, Jian Zheng, Ying Yang

**Affiliations:** 1 Department of Biotechnology, Beijing Institute of Radiation Medicine, Beijing, 100850 China; 2 Institute for Control of Chinese Traditional Medicine and Ethnic Medicine, National Institutes for Food and Drug Control, Beijing, 100050 China; 3 College of Life and Environmental Sciences, Minzu University of China, Beijing, 100081 China

**Keywords:** Genome sequencing, *Ophiocordyceps sinensis*, Retrotransposons, Genome inflation, Fungal evolution

## Abstract

**Background:**

*Ophiocordyceps sinensis*, which is only naturally found in the high-elevation extreme environment of the Tibetan Plateau, has been used in traditional Chinese medicine. Information concerning the evolutionary and geologic context of *O. sinensis* remains limited, however.

**Methods:**

We constructed the high-quality genome of *O. sinensis* and provided insight into the evolution and ecology of *O. sinensis* using comparative genomics.

**Results:**

We mapped the whole genome of the anamorph/asexual form *Hirsutella* of *O. sinensis* using Illumina and PacBio sequencing technologies and obtained a well assembled genome of 119.2 Mbp size. Long-read Single Molecule Real Time (SMRT) sequencing technology generated an assembly with more accurate representation of repeat sequence abundances and placement. Evolutionary analyses indicated that *O. sinensis* diverged from other fungi 65.9 Mya in the Upper Cretaceous, during the uplift of the Tibetan Plateau. Gene family expansions and contractions in addition to genome inflation via long terminal repeat (LTR) retrotransposon insertions were implicated as an important driver of *O. sinensis* divergence. The insertion rate of LTR sequences into the *O. sinensis* genome peaked ~ 30–40 Mya, when the Tibetan Plateau rose rapidly. Gene Ontology (GO) enrichment analysis suggested that *O. sinensis* contained more genes related to ice binding compared to other closely related fungi, which may aid in their adaptability to the cold Tibetan Plateau. Further, heavy metal resistance genes were in low abundance in the *O. sinensis* genome, which may help to explain previous observations that *O. sinensis* tissues contain high levels of heavy metals.

**Conclusions:**

Our results reveal the evolutionary, geological, and ecological context for the evolution of the *O. sinensis* genome and the factors that have contributed to the environmental adaptability of this valuable fungus. These findings suggest that genome inflation via LTR retrotransposon insertions in *O. sinensis* coincided with the uplift of the Tibetan Plateau. LTRs and the specific genetic mechanisms of *O. sinensis* contributed to its adaptation to the environment on the plateau.

## Background

*Ophiocordyceps sinensis*, or caterpillar fungus, has been referred to as “soft gold” in China due to its medicinal benefits and high cost (up to $12,500 USD/kg) [1–3]. *O. sinensis* has a unique growth environment and exhibits highly-specific growth conditions, resulting in its classification as an endangered resource as well as its sharply rising price [4, 5]. *O. sinensis* only grows at high altitudes ranging from 3000 to 5000 m above sea level in alpine meadows of the Tibetan Plateau [3]. The Tibetan Plateau features numerous characteristics that result in an especially harsh growing environment for plants and other life [6] including extreme cold, low oxygen availability, large diurnal temperature shifts, and strong ultraviolet light exposure. Consequently, *O. sinensis* is only found in four global locales: China, Nepal, Bhutan, and India, although its Chinese populations account for the vast majority of its distribution [7]. Evolutionary analyses indicated that *O. sinensis* diverged from other fungi 65.9 Mya in the Upper Cretaceous, during the uplift of the Tibetan Plateau. The rise of the Tibetan Plateau is a particularly significant event in the evolutionary history of the fungus, in which multiple geological stages were involved [8]. *O. sinensis* only thrives in the Tibetan Plateau region, and as such, is difficult to cultivate due to its peculiar growth characteristics and adaptations to the high-altitude environment of the plateau. The details of adaptation for this significant economic and medicinal resource to its unique environment and how *O. sinensis’* extant distribution relates to the uplift of Tibetan Plateau are still unclear [9, 10], but warrants additional study in order to provide evolutionary and historical context that could inform resource preservation and cultivation efforts.

We performed detailed genomic analyses and high-quality genomic reconstruction of *O. sinensis* using Illumina and PacBio sequencing technologies and analyzed these results in the context of the rise of the Tibetan Plateau and the adaptations that have allowed *O. sinensis* to thrive in this harsh environment. With traditional Chinese medicine receiving increased attention in recent years, some previous studies have examined the genome and transcriptome, as well as the mitochondrial and fungal communities of *O. sinensis*. None of this research, however, has revealed the relationship between geological changes occurring on the Tibetan Plateau and the divergence time of *O. sinensis* [11–17]. Our robust genomic analyses resulted in high quality *O. sinensis* genome, in particular overcoming the hindrance of repeat sequences that leads to incompletely assembled genomes and difficulties in interpreting fungal genomes. As a result, we obtained a well assembled genome of 119.2 Mbp size using Illumina and PacBio sequencing technologies and found that the genome primarily consists of LTR retrotransposons to a much greater degree than its closest relatives. Our evolutionary genetic analyses indicate that, in terms of evolutionary distance, *O. sinensis* is closest to its sister species *Hirsutella minnesotensis*. Based on fossil evidence, we estimate that the differentiation and divergence of *O. sinensis* into its peculiar niche began during the first stage of the Tibetan Plateau rise during the Cretaceous-Paleogene transition (65.9 Mya).

From our analyses, we hypothesize that the period when the Tibetan Plateau rose quickly from ~ 45 to 20 Mya was a critical time for the evolution of the unique *O. sinensis* genome. During this period, numerous retrotransposons and other novel genes were introduced into the *O. sinensis* genome, which likely played an important role in its adaptation to the Tibetan Plateau environment [9, 10]. Gene Ontology (GO) enrichment analysis suggested that *O. sinensis* contains more genes related to ice binding than other closely related fungi, which may have enhanced its adaptability to the cold Tibetan Plateau. Further, heavy metal–resistant genes were found to be in low abundance in the *O. sinensis* genome, which may help to explain previous observations that *O. sinensis* tissues contain high levels of heavy metals. Taken together, our results provide an evolutionary and adaptive framework to inform the cultivation and preservation efforts of this rare economic and medicinal fungal resource.

## Materials and methods

### Fungal strain acquisition

*O. sinensis* strain CC1406-203 (catalogued at the National Institutes for Food and Drug Control, China) was isolated from the stromal tissue of a fruiting-body originally collected from the Naqu area in the Tibet autonomous region.

### DNA extraction and sequencing

*O. sinensis* was grown on potato dextrose agar (PDA) at 18 °C for 60 days. Genomic DNA for whole-genome sequencing was extracted from pure cultures using the DNeasy Plant Mini kit (QIAGEN Co., Ltd, Hamburg, Germany) according to the manufacturer’s instructions. We used a whole genome sequencing strategy that incorporated both Illumina MiSeq (Illumina Inc., San Diego, CA, USA) and Pacific Biosciences RS II sequencing platforms (Pacific Biosciences, Menlo Park, CA, USA). The Illumina DNA library (400-bp fragments) was prepared using a TruSeq DNA sample prep kit (Illumina Inc., San Diego, CA, USA) as specified by the manufacturer’s instructions and then sequenced using a MiSeq paired-end 2 × 250 bp (PE250) cycle sequencing kit. Pacific Biosciences RS II sequencing was performed in order to obtain long reads of gDNA sequencing and increase genome coverage. A 10 kbp SMRT bell library was prepared from sheared genomic DNA using a 10 kbp template library preparation workflow. SMRT sequencing was conducted on the PacBio RS II sequencing platform using C3 sequencing chemistry and a P5 polymerase with 32 SMRT cells.

### Genome assembly

De novo assembly of PacBio sequences was carried out using continuous long reads (CLR) following the Hierarchical Genome Assembly Process (HGAP) workflow (PacBioDevNet; Pacific Biosciences) as available in the SMRT Analysis software package v.2.3. The HGAP workflow consists of preassembly, de novo assembly with the Celera Assembler (CA), and assembly polishing with Quiver. Before assembly with the CA software (v.7.0), the PacBio Rs_PreAssembler.1 module was used to perform error correction of the raw data specifying a default minimum subread length of 500 bp, a minimum read quality of 0.80, and a minimum subread length of 7500 bp. MiSeq reads were mapped to the PacBio error-corrected assemblies using the BWA alignment software v0.7.5a. SNPs and small insertions and deletions (INDELs) were then called and corrected using samtools v0.1.18 and in-house scripts.

### Gene prediction and annotation

Coding gene prediction was performed using AUGUSTUS v. 3.1 [18] to produce high confidence gene models. All of the predicted gene models were functionally annotated based on sequence similarity to genes and proteins in the NCBI nucleotide (Nt), non-redundant and UniProt/Swiss-Prot protein databases. Gene models were also further annotated based on protein domains using InterProScan. All genes were classified according to GO, eukaryotic orthologous groups (KOG) and the Kyoto Encyclopedia of Genes and Genomes (KEGG) metabolic pathways. Repeat sequences were masked throughout the genome using Repeat Masker (v. 3.2.9) and the RepBase library (v. 16.08).

### Detection and classification of repetitive elements

Repetitive elements were identified using RepeatModeler (https://www.repeatmasker.org/RepeatModeler.html), and consensus sequences were mapped using RepeatMasker (https://www.repeatmasker.org). Transposable elements were further analyzed using the LTRharvest v. 1.5.7 [19], LTR_Finder v. 1.0.6 [20], and TEclass v. 2.1.3 [21] software packages.

### Orthology and phylogenomic analysis

Homologous gene families were identified using OrthoMCL and gene families that contained only a single gene in the genome (single-copy gene families) were then extracted. For each single-copy gene family, proteins of homologous genes (identified by protein database searches) were aligned using MUSCLE [22]. Concatenated sequences were subjected to Maximum-Likelihood phylogenetic analyses with the RAxML software package v. 8.0.2 [23] specifying a PROTGAMMAJTT substitution model and 1000 bootstrap replicates to assess confidence in tree topologies. The MCMCtree program in the PAML package v4.9e [24, 25] was used to estimate divergence time based on DNA sequences. The divergent times of *O. unilateralis* and *O. sinensis*, *Aspergillus fumigatus* and *Aspergillus niger*, and *Fusariem graminearum* and *Escovopsis weberi* were used as calibration time, which were downloaded from the TimeTree database (. Retrotransposon divergence times in *O. sinensis* were estimated with the formula ${\text{T = K/2r}}$, where K is the number of synonymous substitutions per synonymous site (Ks), and ${\text{T = K/2r}}$ r represents a substitution rate of 1.02 × 10^−9^ per site per year based on previously published estimates [27, 31].

### Gene family expansions and contractions

Gene family expansions and contractions were explored using the Computational Analysis of gene Family Evolution (CAFE) [32]. Candidate expansion and contraction genes were then compared to the GSE52425 Tibetan Plateau soil GeoChip dataset [33] and secondary metabolite results. The Tibetan soil GeoChip data was selected for comparison because *O. sinensis* is naturally found in Tibetan soils, and the gene array in GSE52425 focuses predominantly on alpine environment adaptability, which is consistent with our motives for investigation. The enrichment of the expansion and contraction of candidate gene families was assessed using Fisher’s exact test.

### RIP mutation and DNA methylation analysis

Repeat-induced point (RIP) mutated regions were identified using RIPCAL v2.0 based on high values of TpA/ApT (> 1.61) and low ratios of (CpA + TpG) / (ApC + GpT) (< 0.53) (Additional file 1. Fig. S1) [21]. To assess DNA methylation patterns, the epigenetic modification module in the SMRT portal software was used to analyze the methylation state of the assembled genome.

### Comparative genomics

To identify genes that were enriched in *O. sinensis*, we performed GO enrichment analysis using Fisher's exact test. Genomes from *O. sinensis* CC, *Cordyceps militaris* CM01, *Hirsutella minnesotensis* 3608, *O. unilateralis*, *Metarhizium anisopliae* ARSEF23, *Metarhizium acridum* CQMa102, and *Beauveria bassiana* ARSEF2860 were compared against the GO database and the GSE52425 GeoChip data set [33] specifying thresholds of sequence identity as ≥ 30, ≥ 50% coverage of comparison area, and a *p* value filtering criterion of ≤ 1 × 10^−10^. Statistical comparisons of different functional categories were used to assess gene enrichments in *O. sinensis* relative to other closely related fungi species.

In order to identify the genes related to pathogenicity, the protein sequences of *O. sinensis* CC1406-203, *Cordyceps militaris* CM01, *Hirsutella minnesotensis* 3608, *O. unilateralis*, *Metarhizium anisopliae* ARSEF23, *Metarhizium acridum* CQMa102, and *Beauveria bassiana* ARSEF2860 were then compared against the PHI dataset using BLASTp homology comparison, specifying thresholds of sequence identity as ≥ 50, ≥ 50% coverage of the comparison area and a *p* value filtering criterion of ≤ 1 × 10^−10^. (The PHI-base pathogen-host interactions and amino acid sequences of the PHI-base accessions in FASTA format were downloaded from https://www.phi-base.org/.) Highly homologous genes from *Aspergillus nidulans* FGSC A4 that were unrelated to parasitic or pathogenic physiologies were removed. Lastly, candidate PHI genes from *O. sinensis* were compared to those of related fungi to assess whether they were *O. sinensis* specific and/or PHI genes that were lost in the divergence of *O. sinensis*.

## Results

## Genome sequencing and assembly

Whole genome sequencing of *O. sinensis* CC1406-203 was performed using a shotgun sequencing strategy and the combination of Illumina MiSeq and PacBio sequencing platforms. Illumina MiSeq sequencing generated 2.7Gbp of raw sequence data using a paired-end sequencing library with 400 bp fragment inserts. An additional 7.5 Gbp of sequencing data was generated with PacBio RS II sequencing. The total sequence coverage of the whole genome assembly was 85.6 × .

Using the HGAP assembly pipeline, we combined and assembled Illumina and PacBio sequencing data to obtain a composite 119.2 Mbp genome. The final assembly comprised 1685 contigs, in which the largest contig and N50 were 1.8 Mbp and 326.7 kbp, respectively. The genome integrity was much higher than what had been previously published for an earlier *O. sinensis* genome [11, 17]. The considerably higher quality of the data allowed for more accurate functional and comparative genomic analyses. Additional genomic assembly characteristics are provided in Table 1. The *O. sinensis* CC1406-203 genome was deposited into GenBank under BioProject PRJNA386225, accession number NGJJ00000000.

**Table 1:** Genome assembly characteristics for *Ophiocordyceps sinensis*

Genomic feature	*O. sinensis*
Sequencing depth (fold)	85.6
Genome size (Mbp)	119.2
Contigs	1685
Largest contig (Mbp)	1.8
N50 length (kbp)	326.7
GC content (%)	44.7
Coding GC content (%)	61.0
Predicted rRNAs	37
Predicted tRNAs	156
Repetitive sequences (%)	81.5
Coding region (%)	12.6
Predicted genes	8621
Average gene size (kbp)	1.7
Average exon number per gene	2.8
Average gene exon length (bp)	539.3
Average gene intron length (bp)	112.3

### Gene prediction and functional annotation

Using genomic and transcriptomic data, we performed gene annotation on the *O. sinensis* genome assembly. Results indicated that the genome contained 8621 protein-coding genes (gene models, Additional file 2. Table S1), 37 predicted rRNA genes (Additional file 3. Table S2) and 156 predicted tRNA genes (Additional file 4. Table S3). Average gene length was 1.7 kbp, with 2.8 average exons per gene, and an average exon and intron length of 539 bp and 112 bp, respectively. The size of the coding region was 15.0 Mbp, which comprised 12.6% of the whole genome, in addition to 81.5% that consisted of repeat sequences. Genomic G + C content was 44.7% while the proportion of GC in coding regions was 61.0% (lower GC ratios in repeat regions represent lowered DNA stability). The total coding region size in *O. sinensis* was close to that of the total intron length. These results indicated that the significantly larger genome size of *O. sinensis* relative to other fungi is a result of a larger proportion of repeat sequences. Further gene modeling statistics are provided in Table 1. BUSCO (version: 3.0.2) [34] was then used to evaluate the quality of the gene annotation. The values of core gene estimation were calculated as follows: C: 93.7% [S: 92.7%, D: 1.0%], F: 3.0%, M: 3.3%, n: 303, where C, S, D, F, M and n indicated complete BUSCOs (Benchmarking Universal Single-Copy Orthologs), complete and single-copy BUSCOs, complete and duplicated BUSCOs, fragmented BUSCOs, missing BUSCOs and total BUSCO groups searched, respectively. The results indicate that the gene annotation covered most genetic regions, further confirming the quality of the gene annotation.

### Genome evolution and divergence time estimates

**Fig. 1:**
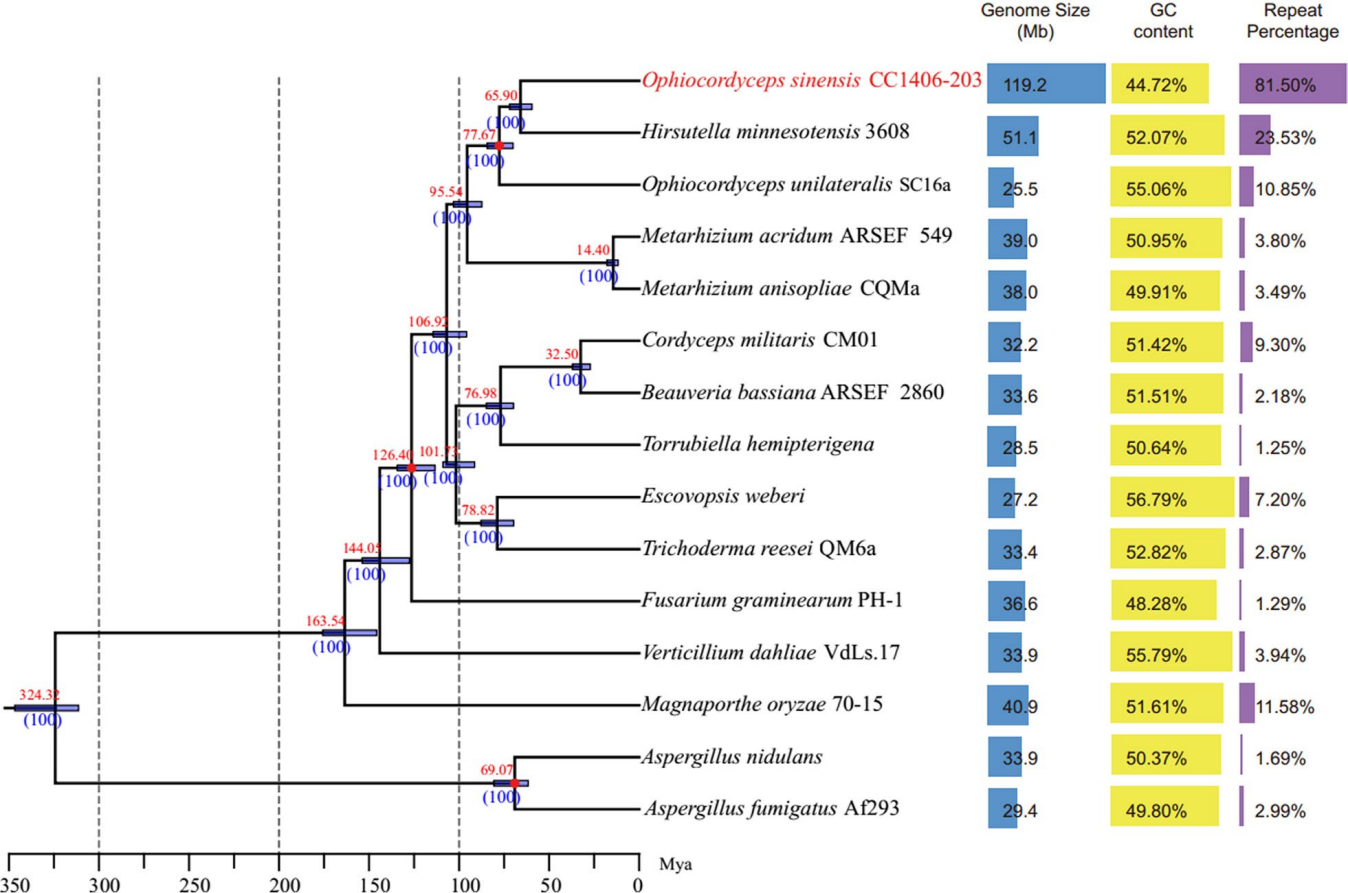
Phylogenetic analyses and comparison of genomic features between *Ophiocordyceps sinensis* and other fungi. The phylogenetic tree is shown on the left based on 2629 single-copy orthologous gene families. Divergence dates, as estimated using MCMCtree software, are provided at nodes. the divergence time of *O. sinensis* and its sister species H. minnesotensis to be approximately 65.90 Mya. The genomic features are provided on the right for each genome (GC content and percentage of genome that is comprised of repeat sequences). Divergence time estimates (in Mya) and 95% confidence intervals for nodes are presented as black digits and blue bars, respectively. The red dots indicate the divergent time used for recalibrations

In order to examine the evolutionary relationships of *O. sinensis* with other closely related fungi, the protein sequences of 15 species were analyzed. A total of 2629 single-copy orthologous genes (Additional file 5. Table S4) were selected for further alignment using MUSCLE (v. 3.6) and then concatenated into a single multiple-sequence alignment using an in-house Perl script. A maximum likelihood phylogeny was reconstructed using RAxML (Fig. 1). The phylogenetic analyses indicated that *O. sinensis* and its congeneric species *Hirsutella minnesotensis* are sister groups. Based on the phylogeny and fossil records, we dated the divergence time of *O. sinensis* and *H. minnesotensis* to be approximately 65.90 Mya.

### Repetitive elements in the *Ophiocordyceps sinensis* genome

**Fig. 2:**
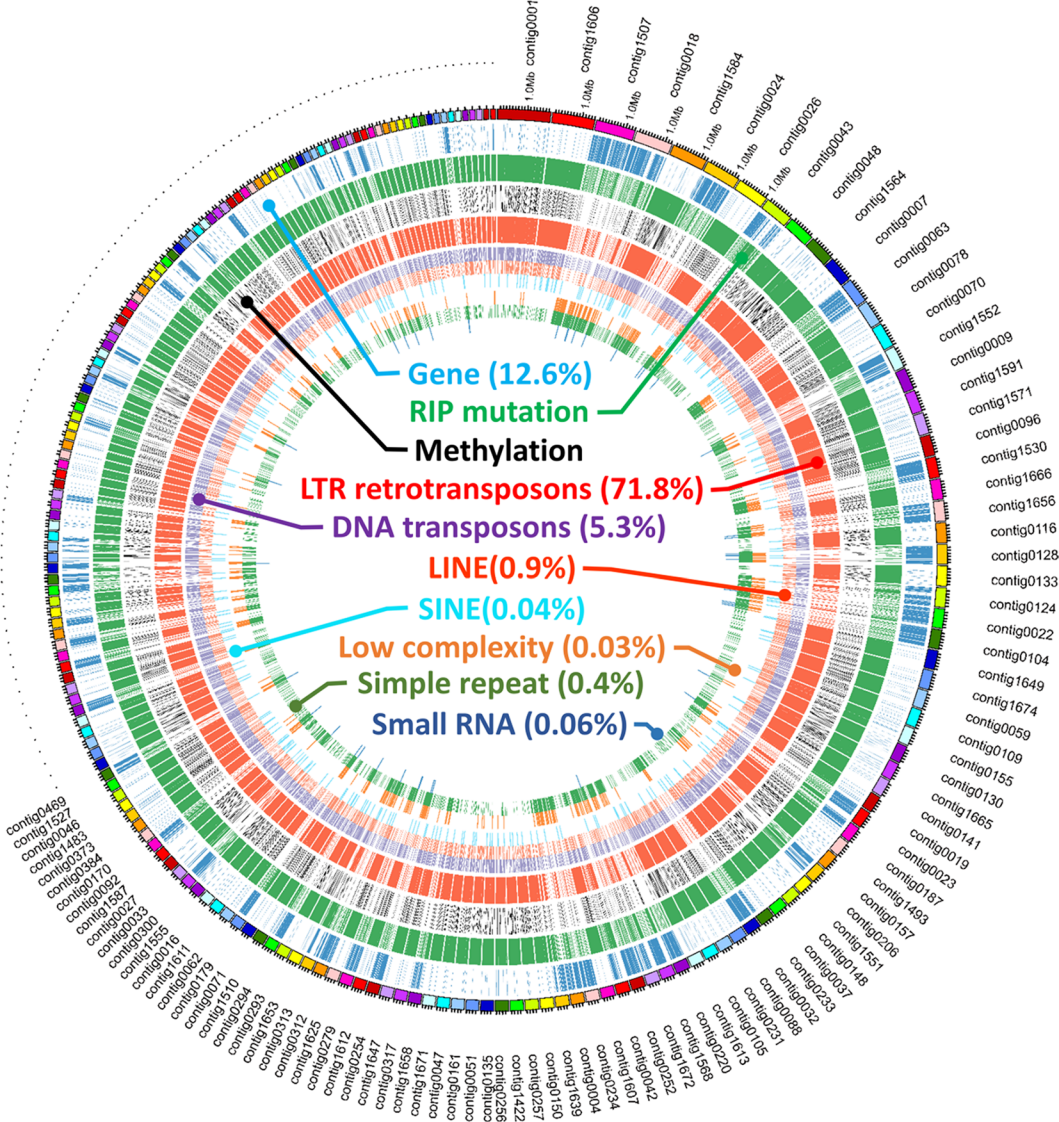
Distribution map of genomic characteristics within the *Ophiocordyceps sinensis* genome. The outer ring represents the first 200 contigs in the genome of *O. sinensis. *Rings (outside to inside) correspond to: the position of genes (blue), repeat-induced point (RIP) mutation positions (green), methylated regions (black), LTR sequence positions (red), DNA transposons (purple), long interspersed repeat sequences (LINEs; light red), short interspersed repeat sequences (SINEs; light blue), low complexity sequences (brown), simple tandem repeat sequences (dark green), and small RNA sequences (dark blue). Percentages in parentheses represent the contribution of each element to the whole genome

Predictions of repeat regions within the *O. sinensis* genome indicated that 81.5% of the genome comprised repeat sequences. Of that genomic portion, retrotransposons comprised 88.1% of the total repeat sequence. The distribution and proportion of main repeat elements in the *O. sinensis* genome are shown in Fig. 2 and all the repeat elements are listed in Additional file 6. Table S5.

Long terminal repeat (LTR) elements represented the majority (71.8%) of the genome. Other major elements consisted of genic regions (12.6%), DNA transposons (5.3%), long interspersed repeat elements (LINEs; 0.9%), short interspersed repeat elements (SINEs; 0.04%), low-complexity sequence (0.03%), simple tandem repeat sequences (0.4%), and small RNA sequences (0.06%). Low complexity sequence and simple tandem repeat sequence shared similar distribution patterns (Fig. 2), while the distribution of the Repeat-induced point (RIP) mutation regions and methylated regions were coincident with the distribution of LTR elements (Additional file 7. Table S6 and Additional file 8. Table S7, Additional file 1. Fig. S1).

The distribution of LTR retrotransposons and gene regions were mutually exclusive in the *O. sinensis* genome. We further analyzed the distribution of RIPs and DNA methylation states, which both affect the transposition function of LTR retrotransposons. RIP mutations provide a defense mechanism for eukaryotic organisms against transposons by generating non-synonymous mutations on susceptible repeat sequence regions that de-functionalize transposons. In addition, DNA methylation can also inhibit LTR retrotransposon functionality via suppression. RIP mutations, methylated regions and LTR elements were all located in non-coding regions, and their distribution patterns were equivalent (Fig. 2).

### LTR retrotransposon analysis

A total of 61,748 LTR retrotransposon sequences were predicted, comprising a total of 85.5 Mbp (Additional file 9. Table S8). 5996 LTR retrotransposons were identified as having complete LTR sequences at both sequence ends, with an average length of 5.9 kbp and comprising 35.1 Mbp total sequence length. The abundance of predicted LTR elements is consistent with previous studies [31, 32]. LTR retrotransposons primarily included Ty3/Gypsy and Ty1/Copia type LTR retrotransposons; with each accounting for 45.9 and 42.2% of the total LTR sequence, respectively. Detailed LTR retrotransposon sequence type abundances are listed in Table 2.

**Table 2:** Categories and abundances of LTR types

	Number	Length (kbp)
LTR.Copia	27,699	36,125.849
LTR.ERV1	269	172.594
LTR.Gypsy	26,672	39,230.793
LTR.Ngaro	147	186.706
LTR.Pao	143	15.967
Unknown	6818	9803.887
Total	61,748	85,535.796

**Fig. 3:**
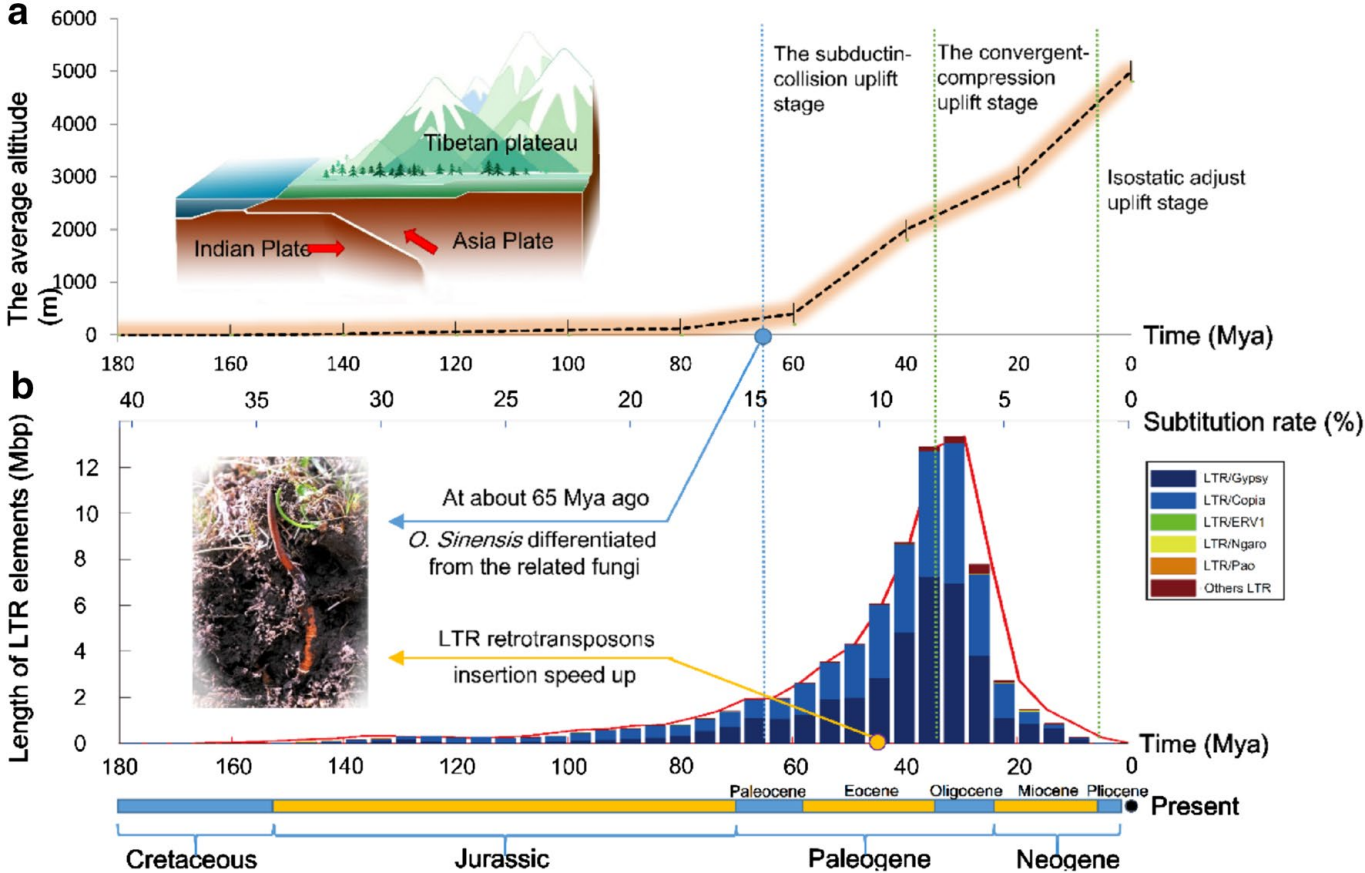
Correspondence between the geological formation of the Tibetan Plateau and the diversity of LTR elements in the *Ophiocordyceps sinensis* genome. **a** Collision between the Indian and Asian continental plates resulted in the uplift of the Tibetan Plateau. **b** Stacked bars indicate the total length of the LTR element types (y-axis), organized by their estimated substitution rate (x-axis). The red line indicates the estimated origin date for each set of LTR sequences

Using LTR sequences that had been identified by RepeatModeler, we further analyzed their differentiation among our fungal genome dataset and estimated differentiation times based on these data (Fig. 3). Results indicated that LTR element differentiation primarily occurred in the Cenozoic Paleogene period (~ 65–23 Mya), when the Tibetan Plateau began to rise.

LTR transposons have been shown to insert themselves into gene coding regions in animal, plant and fungal genomes, thus affecting gene function and regulation of adjacent gene expression. We selected entire LTR sequence regions together with sequences that were 1 kbp upstream and downstream in order to assess overlap with gene coding regions. We then selected the ten most significantly enriched genes with known function and compared their enrichment to the genome background using Fisher’s exact test (Table 3). The analyses suggested that LTR retrotransposons primarily affected regions that included genes such as Mannuronan C-5-epimerase and Phosphomannomutase. The enrichment of a Zinc finger domain-containing protein may indicate an effect on gene regulation and subsequent regulation of *O. sinensis* adaptability. The enrichment of a TY/3B-TY3B protein is likely related to the presence of the most abundant LTR element in the *O. sinensis* genome, Ty3/Gypsy.

**Table 3:** Gene family enrichment in LTR regions

Gene family	Count^a^	*p* value
Zinc finger domain-containing protein	148	2.20 × 10^−16^
Mannuronan C-5-epimerase C-terminal fragment	57	2.20 × 10^−16^
Polyprotein	39	1.32 × 10^−11^
TY3B-TY3B protein	29	9.79 × 10^−09^
Phosphomannomutase	27	1.82 × 10^−10^
Aggrecan core protein	26	4.48 × 10^−10^
Endoplasmic reticulum oxidoreductin 1	26	3.91 × 10^−09^
PREDICTED: NFX1-type zinc finger-containing protein	26	3.91 × 10^−09^
PREDICTED: filaggrin-2	22	1.63 × 10^−08^
PREDICTED: keratin-associated protein 10–11-like	22	1.23 × 10^−07^

^a^Count represents the number of genes that overlap between gene coding regions and the LTR sequence region or 1 kbp upstream/downstream from LTR

### Functional gene enrichment analysis

**Fig. 4:**
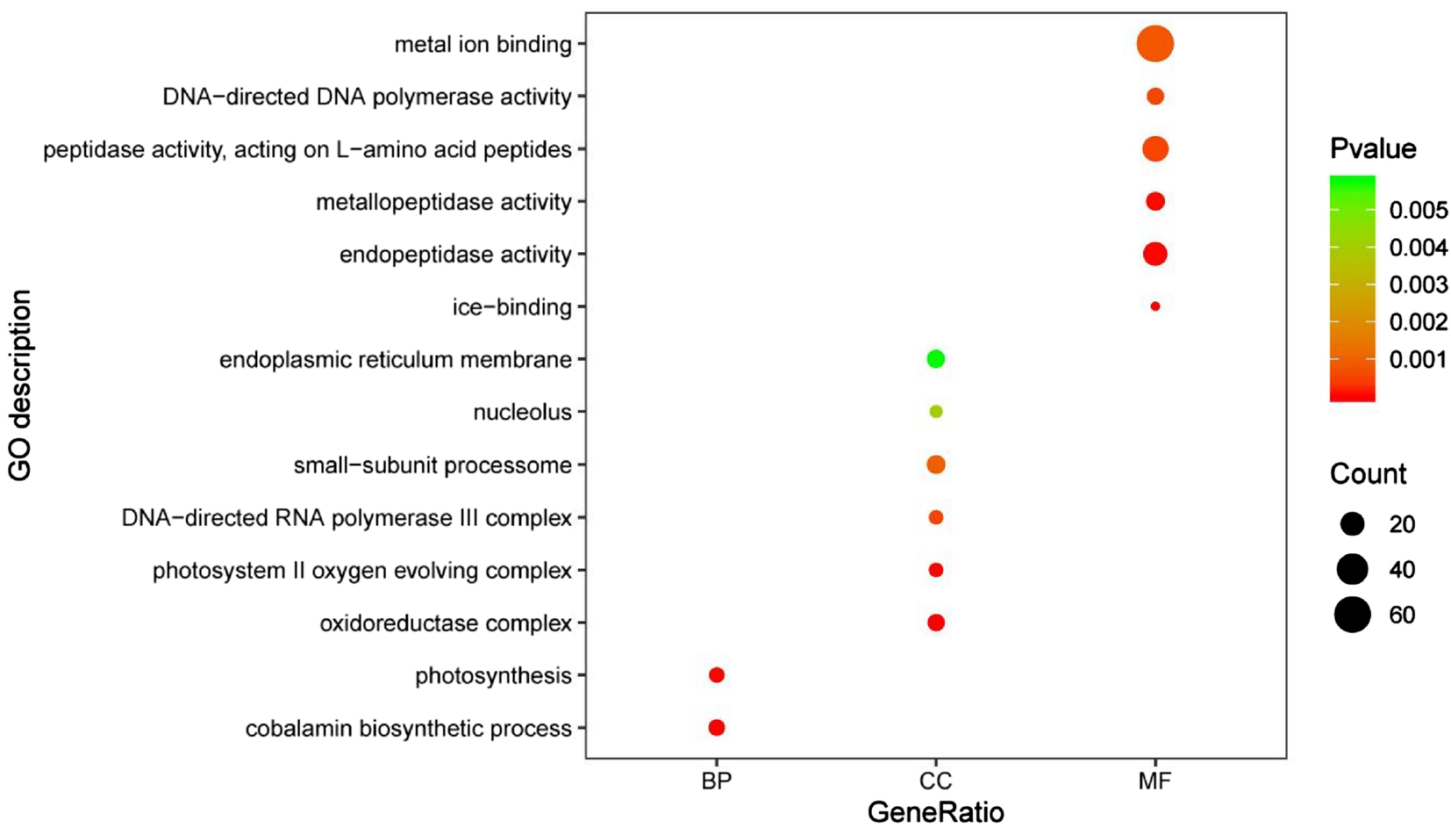
GO function differences among *Ophiocordyceps sinensis* and other closely related fungi. Fourteen functional GO terms that were statistically different among 7 background genomes were identified. CC, MF, and BP denote 3 categories of GO function: *CC* cellular component, *MF* , molecular function, and *BP* biological process

In order to identify the enrichment of specific functions in the genome of *O. sinensis*, we compared it to other closely related fungi, including *Hirsutella minnesotensis*, *O. unilateralis*, *Beauveria bassiana*, *Cordyceps militaris*, *Metarhizium acridum*, and *Metarhizium anisopliae*. Differential GO function enrichment analysis indicated that 14 GO categories were significantly different (*p* < 0.05 by multi-group chi-square test) among these 7 genomes (Fig. 4). The GO terms associated with DNA-directed DNA polymerase activity (GO:0003887), endopeptidase activity (GO:0004175), ice-binding (GO:0050825), metal ion–binding (GO:0046872), metallopeptidase activity (GO:0008237), peptidase activity, acting on L-amino acid peptides (GO:0070011), DNA-directed RNA polymerase III complex (GO:0005666), endoplasmic reticulum membrane (GO:0005789), nucleolus (GO:0005730), oxidoreductase complex (GO:1990204), photosystem II oxygen-evolving complex (GO:0009654), small-subunit processome (GO:0032040), cobalamin biosynthetic process (GO:0009236), and photosynthesis (GO:0015979) were enriched in all categories (Additional file 10. Table S9). These genes, especially ice-binding (GO:0050825) and photosystem II oxygen-evolving complex (GO:0009654) may be related to the high-altitude, low-oxygen, and cold environment found on the Tibetan Plateau. In addition, the metal ion–binding (GO:0046872) may help to explain previous observations that O. sinensis tissues contain high levels of heavy metals.

### Gene expansion and contraction

**Fig. 5:**
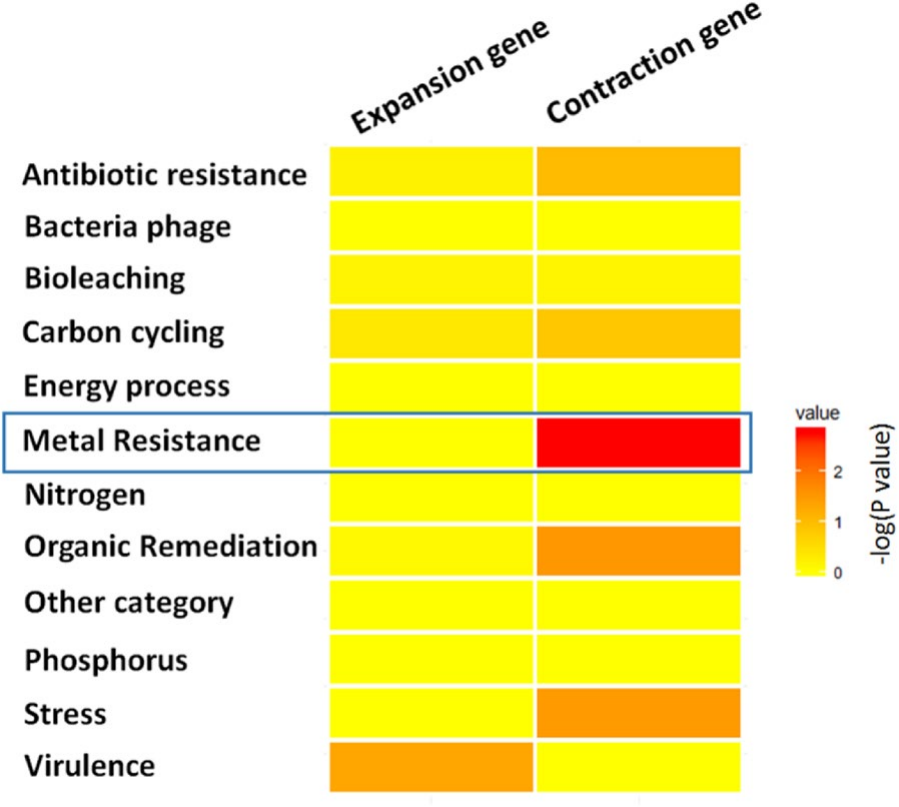
Functional gene families that were contracted or expanded in *the Ophiocordyceps sinensis* genome. Contracted or expanded gene families of *O. sinensis* were identified using CAFE, and the genes were classified using the Tibetan Plateau soil GeoChip data set. Enrichment of functional categories was assessed using Fisher's exact test. Heatmap colors correspond to resultant *P* values according to the legend on the right. The most statistically significant contracted functional category was “Metal Resistance,” which is indicated by a blue box

We compared homologous genes from the entire genome of *O. sinensis* with those of closely related species to identify instances of gene expansion and contraction. In total, 243 genes in *O. sinensis* were in gene families that were expanded while 89 genes were in gene families that were contracted. In subsequent secondary metabolic gene functional analysis, we identified 26 intermediate metabolic genes in the gene expansion subset and two secondary metabolic genes in the gene contraction subset. Enrichment of secondary metabolic genes in the expansion gene subset was significantly higher than that of the *O. sinensis* whole genome background (Fisher’s exact test, P = 2.2 × 10^−16^). The result was not surprising, considering that most of *O. sinensis*’ secondary metabolic genes were in the expansion gene subset. We then compared the expansion/contraction gene family subset against the GSE52425 GeoChip data set [33] to assess their potential function with regards to alpine environment adaptability. The subsets of genes predicted to be contracted were primarily involved in metal resistance (Fig. 5), and one of them was related to heavy metal resistance (Additional file 11. Table S10).

### Host infectivity pathways and medicinally-related secondary metabolite genes

In order to assess changes in infectivity related to the divergence of the caterpillar fungus, we compared *O. sinensis* genes with the pathogen host interaction (PHI) database and 6 other closely related fungi (*H. minnesotensis*, *O. unilateralis*, *B. bassiana*, *C. militaris*, *M. acridum*, and *M. anisopliae*). In total, we found that the *O. sinensis* genome comprised 67 PHI categories shared among 6 closely related fungi (Additional file 12. Table S11), and further identified 3 PHI categories that were unique to *O. sinensis*, as well as 9 PHI categories that were absent in *O. sinensis*. Among the 9 PHI categories absent in *O. sinensis*, the one with highest nucleotide similarity was PHI:409, which represents a chitinase that can confer enhanced toxicity and cuticle degradation of host insects under pathogenic circumstances (Additional file 12. Table S11) [35]. Loss of this specific gene suggests a lesser infectivity potential of *O. sinensis* on insect hosts. We also identified 68 secondary metabolic genes in *O. sinensis* and 3 of the metabolic genes were only present in *O. sinensis* when comparing against the other closely related fungi (Additional file 13. Table S12).

## Discussion

*O. sinensis*, the psychrophilic, slow-growing, and valuable medicinal fungus, only grows on the Tibetan Plateau at elevations ranging from 3–5 km above sea level under the extreme conditions present there [2]. As a consequence of a highly specific and extreme environmental niche, *O. sinensis* has resisted efforts for efficient cultivation, which has exacerbated the lack of supply to meet market demands [3, 4]. The significant economic and scientific value of *O. sinensis* has led to increased attempts to better understand its physiologic properties, which could inform conservation and cultivation efforts. Here, we have constructed a high-quality genome of *O. sinensis* and have used comparative genomics to contextualize the evolutionary history and ecological characteristics of the fungus.

The data presented here build on previous genomic reconstructions for *O. sinensis* [11], but provide a more robust and complete genome that was assembled using a multi-platform sequencing approach to achieve a higher-quality assembly. In particular, our use of long-read PacBio sequencing technology allowed us to generate an assembly with more accurate representation of repeat sequence abundance and placement. Consequently, our results indicate that *O. sinensis* has undergone genome expansion due to an increased incorporation of repeat sequences, and particularly retrotransposons, into its genome. Our analyses also suggested that RIP mutations and DNA methylation states were highly coincident with these repeat regions. Since RIPs can suppress transposition activity and methylation can effectively silence their activity, these results suggest that the fungus has evolved to mitigate deleterious effects arising from rampant transposon insertion via these mechanisms. Using the single-copy orthologous genes to determine the divergence times between *O. sinensis* and closely related species, our phylogenetic analyses estimated that *O. sinensis* began to diverge from its closest ancestor (with an available genome) 65.9 Mya which corresponds to the late Cretaceous or early Cenozoic period. Recent models of the Tibetan Plateau formation suggest that the first phase of the rise began ~ 44 Mya [36], which is generally in agreement with our estimates for *O. sinensis* divergence times. We hypothesize that *O. sinensis* began to diverge from other lower-elevation adapted fungi at the onset of the Tibetan Plateau rise and that this divergence was, in part, associated with genome inflation due to LTR retrotransposon insertions. During the Cretaceous period (~ 140–60 Mya), when the Tibetan Plateau had yet to be formed, the abundance of repeat sequences in the *O. sinensis* genome remained at low levels. However, during the Upper Cretaceous, when the subduction of the Indian plate to the Eurasian plate occurred and resulted in the uplift of the Tibetan Plateau [37, 38], LTR sequences began to accumulate at an accelerated rate in the *O. sinensis* genome. Further, when the Tibetan Plateau began to uplift considerably 30–40 Mya, the insertion rate of LTR sequences into the *O. sinensis* reached peak values. Finally, when the elevation of the Tibetan Plateau reached ~ 5 km, the abundance of LTR sequences in the *O. sinensis* genome stabilized and has remained relatively unchanged.

Analysis of genes flanking the LTRs in the *O. sinensis* genome revealed that LTR retrotransposons were especially associated with Mannuronan C-5-epimerase and Phosphomannomutase that are involved in d-mannose synthesis, which itself is one of the primary components of *O. sinensis* ascocarp polysaccharides [39]. Thus, a plausible hypothesis from these results is that the adaptation to a lower temperature, high elevation, extreme environment was, in part, due to retrotransposon-mediated alteration of structural polysaccharide gene abundances. However, further studies are needed to assess whether LTR retrotransposon insertions may also contribute to other effects on *O. sinensis* that are currently unknown.

We speculated that *O. sinensis* may harbor unique secondary metabolism genes due to its divergence and adaptation to the extreme environment of the Tibetan Plateau. Intriguingly, enrichment of secondary metabolite gene families was evident when comparing *O. sinensis* to other closely related fungi, and these families comprised nearly the entire subset of genes that were enriched in the genome (particularly organic remediation gene families). Moreover, when analyzing the families of genes that were expanded based on duplication events, *O. sinensis* secondary metabolite genes comprised nearly the entire expanded gene dataset and the dataset comprised nearly all of the secondary metabolite genes present in the *O. sinensis* genome. GO enrichment analysis indicated that *O. sinensis* contained more genes related to ice binding (GO:0050825) compared to other related fungi, which may confer increased cold adaptability for life in the Tibetan Plateau environment. Of the gene families that we found to be contracted in *O. sinensis* relative to closely related fungi, heavy metal resistance genes were prominent. Previous studies have indicated that *O. sinensis* tissue contains high levels of heavy metals that exceed safety standards [40]. The persistence of heavy metals may be related to the decreased abundances of metal resistance genes and provides context for cultivation studies as it relates to the usage of metals in cultivation media. Further research regarding the specific genes that are present or absent in *O. sinensis* (as we have documented here) relative to other closely-related fungi will aid in cultivation efforts and enhance its practical application value.

Lastly, a critical component of the caterpillar fungus’ lifestyle is the infection of ghost moth larvae in order to produce fruiting bodies [1, 3, 4]. To assess the nature of infectivity that is unique to *O. sinensis*, we surveyed its genes against the pathogen-host interaction (PHI) database to compare *O. sinensis* with closely related fungal species. We found a paucity of PHI-identified genes in *O. sinensis* relative to other fungi, and in particular a lack of chitinase genes. This result may point towards reduced infectivity pathways in *O. sinensis* and ultimately lead to either difficulty in host infection or a highly-specialized mechanism of caterpillar infection. These suppositions are consistent with the relative inability to culture *O. sinensis* artificially, which may also be due in part to its streamlined infection pathways.

## Conclusions

We have constructed the high-quality genome for the fungus *O. sinensis* using a multi-platform sequencing methodology incorporating both Illumina and PacBio sequencing technologies. The high-quality genome allowed us to infer a number of characteristics regarding the genome, evolution and biology of *O. sinensis* which have yet to be reported. Our results suggest that genome inflation via LTR retrotransposon insertions into the *O. sinensis* genome has been an important driver of its divergence from other closely-related fungi and we estimate that these processes occurred in coincidence with the rise of the Tibetan Plateau and contributed to the adaptation of this specialist fungal species to a high-elevation, extreme environment on the plateau. The results reported here provide a critical genomic framework from which to begin assessing which specific cultivation conditions may be necessary to encourage the growth of this specialist fungus, in addition to ecological and evolutionary context to inform conservation efforts.

## Supplementary information

**Additional file 1: Figure S1.** Dinucleotide frequency analysis of *O. sinensis* CC1406-203.

**Additional file 2: Table S1.** Protein-coding genes of *O. sinensis* CC1406-203.

**Additional file 3: Table S2.** Predicted rRNAs of *O. sinensis* CC1406-203.

**Additional file 4: Table S3.** Predicted tRNAs of *O. sinensis* CC1406-203.

**Additional file 5: Table S4.** Single copy orthologous genes.

**Additional file 6: Table S5.** Repetitive elements of *O. sinensis* CC1406-203 identified by RepeatModeler.

**Additional file 7: Table S6.** Repeat-induced point mutation regions.

**Additional file 8: Table S7.** Methylation regions of *O. sinensis* CC1406-203.

**Additional file 9: Table S8.** Final LTR elements list.

**Additional file 10: Table S9.** Results of differential GO function enrichment analysis.

**Additional file 11: Table S10.** Genes relative to metal resistance.

**Additional file 12: Table S11.** PHI analysis.

**Additional file 13: Table S12.** Secondary metabolism gene analysis.

## Data Availability

Supplementary figures and tables are available online.

## Abbreviations

*O. sinensis*: *Ophiocordyceps sinensis*
SMRT: Single-molecule real-time
LTR: Long terminal repeat
GO: Gene Ontology
CLR: Continuous long reads
HGAP: Hierarchical genome assembly process
RIP: Repeat-induced point mutation

## References

[1] Cannon PF, Hywel-Jones NL, Maczey N (2009). Steps towards sustainable harvest of *Ophiocordyceps sinensis* in Bhutan. Biodivers Conserv.

[2] Zhou X, Gong Z, Su Y (2009). Cordyceps fungi: natural products, pharmacological functions and developmental products. J Pharm Pharmacol.

[3] Lo HC, Hsieh C, Lin FY (2013). A systematic review of the mysterious caterpillar fungus *Ophiocordyceps sinensis* in Dong-ChongXiaCao (Dong Chong Xia Cao) and related bioactive ingredients. J Tradit Complem Med.

[4] Stone R (2008). Last stand for the body snatcher of the Himalayas?. Science.

[5] Stone R (2010). Improbable partners aim to bring biotechnology to a Himalayan Kingdom. Science.

[6] Wang CT, Long RJ, Wang QJ (2007). Effects of altitude on plant-species diversity and productivity in an alpine meadow. Qinghai-Tibetan plateau Aust J Bot.

[7] Li Y, Wang XL, Jiao L (2011). A survey of the geographic distribution of *Ophiocordyceps sinensis*. J Microbiol.

[8] Paul T, Xu Z, Françoise R (2001). Oblique stepwise rise and growth of the Tibet Plateau. Science.

[9] Wang C, Zhao X, Liu Z (2008). Constraints on the early uplift history of the Tibetan Plateau. PNAS.

[10] van Hinsbergen DJJ, Lippert PC, Dupont-Nivet G (2012). Greater India basin hypothesis and a two-stage Cenozoic collision between India and Asia. PNAS.

[11] Hu X, Zhang Y, Xiao G (2013). Genome survey uncovers the secrets of sex and lifestyle in caterpillar fungus. Chinese Sci Bull.

[12] Xiang L, Li Y, Zhu Y (2014). Transcriptome analysis of the *Ophiocordyceps sinensis* fruiting body reveals putative genes involved in fruiting body development and cordycepin biosynthesis. Genomics.

[13] Li Y, Hu XD, Yang RH (2015). Complete mitochondrial genome of the medicinal fungus *Ophiocordyceps sinensis*. Sci Rep.

[14] Liu ZQ, Lin S, Baker PJ (2015). Transcriptome sequencing and analysis of the entomopathogenic fungus *Hirsutella sinensis* isolated from *Ophiocordyceps sinensis*. BMC Genomics.

[15] Xia F, Chen X, Guo MY (2016). High-throughput sequencing-based analysis of endogenetic fungal communities inhabiting the Chinese Cordyceps reveals unexpectedly high fungal diversity. Sci Rep.

[16] Zhong X, Gu L, Li SS (2016). Transcriptome analysis of *Ophiocordyceps sinensis* before and after infection of Thitarodes larvae. Fungal Biol.

[17] Xia EH, Yang DR, Jiang JJ (2017). The caterpillar fungus, *Ophiocordyceps sinensis*, genome provides insights into highland adaptation of fungal pathogenicity. Sci Rep.

[18] Stanke M, Diekhans M, Baertsch R (2008). Using native and syntenically mapped cDNA alignments to improve de novo gene finding. Bioinformatics.

[19] Ellinghaus D, Kurtz S, Willhoeft U (2008). LTR harvest, an efficient and flexible software for de novo detection of LTR retrotransposons. BMC Bioinformatics.

[20] Xu Z, Wang H (2007). LTR_FINDER: an efficient tool for the prediction of full-length LTR retrotransposons. Nucleic Acids Res.

[21] Abrusán G, Grundmann N, DeMester L (2009). TE class-a tool for automated classification of unknown eukaryotic transposable elements. Bioinformatics.

[22] Edgar RC (2004). MUSCLE: a multiple sequence alignment method with reduced time and space complexity. BMC Bioinformatics.

[23] Stamatakis A (2014). RAxML version 8: a tool for phylogenetic analysis and post-analysis of large phylogenies. Bioinformatics.

[24] Yang ZH (1997). PAML: a program package for phylogenetic analysis by maximum likelihood. Comput Appl Biosci.

[25] Sudhir K, BlairHedges S (2016). Advances in time estimation methods for molecular data. Mol Biol Evol..

[26] Sung GH, Poinar GO, Spatafora JW (2008). The oldest fossil evidence of animal parasitism by fungi supports a Cretaceous diversification of fungal-arthropod symbioses. Mol Phylogenet Evol.

[27] Kijima TE, Innan H (2010). On the estimation of the insertion time of LTR retrotransposable elements. Mol Biol Evol.

[28] Hedges SB, Marin J, Suleski M (2015). Tree of life reveals clock-like speciation and diversification. Mol Biol Evol.

[29] Kensche PR, Oti M, Dutilh BE (2008). Conservation of divergent transcription in fungi. Trends Genet.

[30] Sharpton TJ, Stajich JE, Rounsley SD (2009). Comparative genomic analyses of the human fungal pathogens Coccidioides and their relatives. Genome Res.

[31] Wei L, Xiao M, An Z (2013). New insights into nested long terminal repeat retrotransposons in brassica species. Mol Plant.

[32] De Bie T, Cristianini N, Demuth JP (2006). CAFE: a computational tool for the study of gene family evolution. Bioinformatics.

[33] Chu H, Wang S, Yue H (2014). Contrasting soil microbial community functional structures in two major landscapes of the Tibetan alpine meadow. MicrobiologyOpen.

[34] Waterhouse RM, Seppey M, Simao FA (2017). BUSCO Applications from quality assessments to gene prediction and phylogenomics. Mol Biol Evol.

[35] Fang WG, Leng B, Xiao YH (2005). Cloning of Beauveria bassiana chitinase gene Bbchit1 and its application to improve fungal strain virulence. Appl Environ Microb.

[36] Wang Y, Zheng J, Zhang W (2012). Cenozoic uplift of the Tibetan Plateau: Evidence from the tectonic-sedimentary evolution of the western Qaidam Basin. Geosci Front.

[37] Allégre CJ, Courtillot V, Tapponnier P (1984). Structure and evolution of the Himalaya-Tibet orogenic belt. Nature.

[38] Li T (1995). The uplifting process and mechanism of the Qinhai-Tibet Plateau. Acta Geosicientia Sinica.

[39] Miyazaki T, Oikawa N, Yamada H (2008). Studies on fungal polysaccharides XX galactomannan of Cordyceps sinensis. Chem Pharm Bull.

[40] Zuo HL, Chen SJ, Zhang DL (2013). Quality evaluation of natural Cordyceps sinensis from different collecting places in China by the contents of nucleosides and heavy metals. Anal Methods.

