# Supplementary figures and images for: Genomic analyses reveal evolutionary and geologic context for the plateau fungus Ophiocordyceps sinensis
Source: Chin Med. 2020 Oct 6;15:107. doi: 10.1186/s13020-020-00365-3 (PMC7542391; doi:10.1186/s13020-020-00365-3)

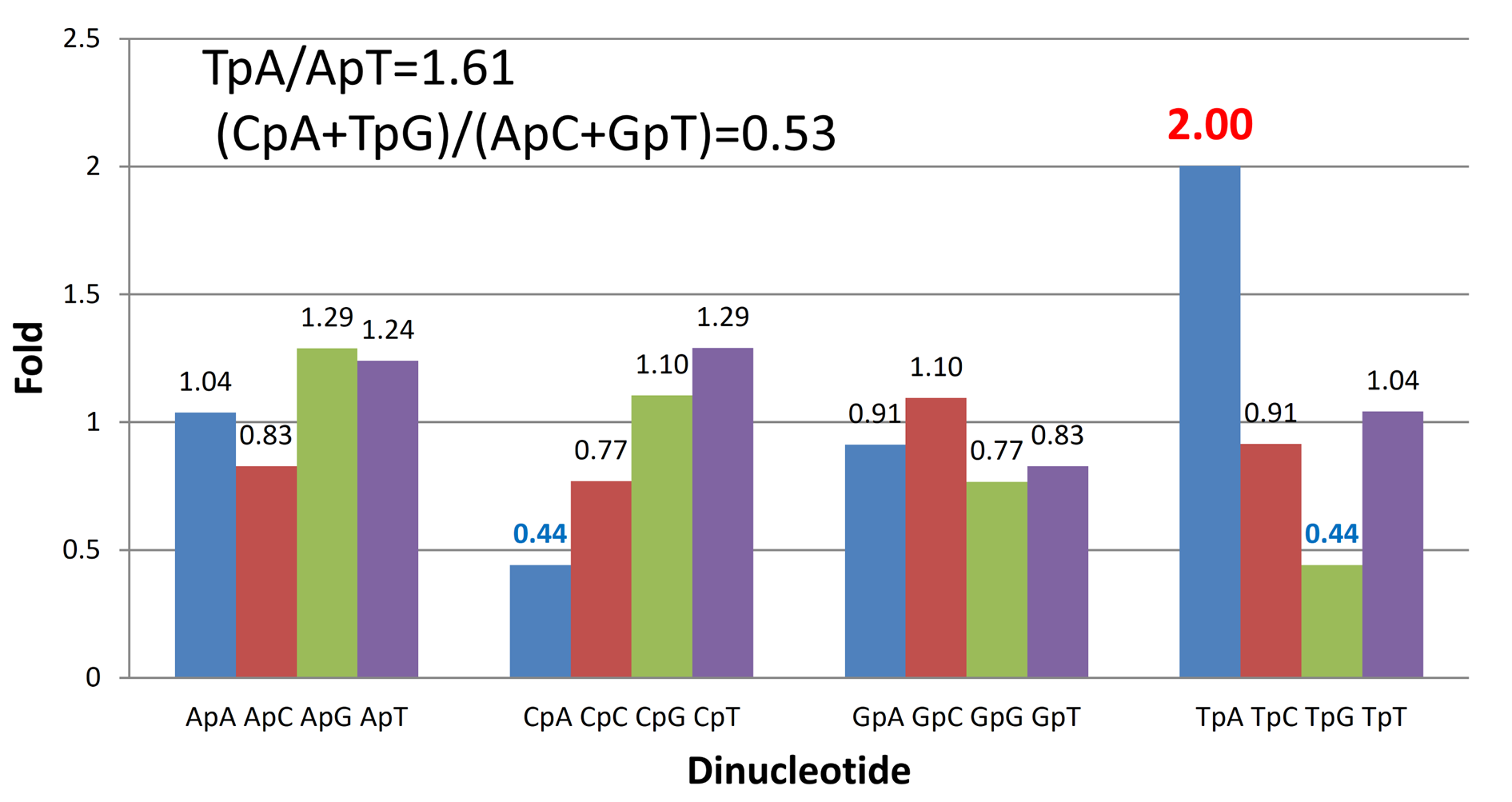

Supplement: Supplementary file 1 — Additional file 1: Figure S1. Dinucleotide frequency analysis of O. sinensis CC1406-203. [file 13020_2020_365_MOESM1_ESM.tif]
